# Intraosseous Metastasizing of Pineoblastoma into the Anterior Skull Base, Calvarial Bones, and Vertebrae

**DOI:** 10.7759/cureus.437

**Published:** 2015-12-28

**Authors:** Denis Golbin, Konstantin V Nikitin, Alexander N Konovalov, David I Pitskhelauri, Liudmila V Shishkina, Andrey V. Golanov, Vasily A Cherekaev, Grigory L Kobiakov, Oksana V Absalyamova, Nikolay Lasunin, Natalia Antipina

**Affiliations:** 1 Skull Base and Craniofacial Surgery, Burdenko Neurosurgery Institute; 2 Department of Radiation Oncology, Burdenko Neurosurgery Institute; 3 Burdenko Neurosurgery Institute; 4 Neurooncology, Burdenko Neurosurgery Institute; 5 Neuropathology, Burdenko Neurosurgery Institute

**Keywords:** pineoblastoma, osseous metastasis, stereotactic radiotherapy, stereotactic radiosurgery, hypofractionated radiotherapy

## Abstract

Pineoblastoma is a rare malignant tumor of the central nervous system (CNS), which arises from the parenchyma of the pineal gland. It is characterized by aggressive clinical behavior and frequent metastases along the craniospinal axis. Extraneural metastases may occur due to surgical seeding of tumor cells beyond the dura and/or hematogenous spread, ventriculoperitoneal shunting, or through Batson’s plexus. To our knowledge, only six documented cases of intraosseous metastases of pineoblastoma are described in the literature.

A 23-year-old female patient presented with clinical and radiological symptoms of a pineal tumor causing secondary hydrocephalus. After initial surgical treatment, chemotherapy, and local radiotherapy with craniospinal irradiation, she developed multiple metastases affecting the anterior skull base, intracranial meninges, frontal bone, and finally, the entire vertebral column. The patient received surgical treatment for the anterior skull base metastasis, repeated irradiation of the neuraxis, radiosurgical and radiotherapeutic procedures, and chemotherapy. The patient survived 57 months after the primary disease manifestation and died of multiple metastases.

This presented case is the first known description of metastasis of pineoblastoma in the anterior cranial base. Multiple intracranial metastases were suppressed using CyberKnife radiation treatment and chemotherapy until massive involvement of spinal column occurred. Interestingly, no signs of brain radiation necrosis after repeated radiation treatments were observed, and the patient developed only moderate neurocognitive decline.

## Introduction

Pineoblastoma is a rare malignant tumor of the central nervous system (CNS), which arises from parenchyma of the pineal gland and primarily affects the pediatric population. Pineal tumors comprise 0.5-1% of all intracranial tumors, and 15-32% of pineal tumors originate from pineal parenchyma. Pineoblastoma represents 25-50% of pineal parenchymal tumors [1]. The lesion is classified as Grade IV, according to the WHO classification of tumors of the CNS. Pineoblastoma is characterized by aggressive clinical behavior and frequent metastases along the craniospinal axis [2]. The most common way of metastasizing is the dissemination of tumor cells via cerebrospinal fluid (CSF); therefore, spinal drop metastases and leptomeningeal metastases are common. Extraneural metastases may occur due to the following reasons: surgical seeding of tumor cells beyond the dura and/or hematogenous spread, ventriculoperitoneal shunting, or through Batson’s plexus [3]. To our knowledge, only six documented cases of intraosseous metastases of pineoblastoma are described in the literature. We present an observation of multiple extraneural osseous metastases of a pineoblastoma, including lesions of the ethmoid roof with intracranial extension, calvarial bones, and numerous foci in the entire spinal column.

Research Involving Human Participants:

All procedures performed in studies involving human participants were in accordance with the ethical standards of the institutional and/or national research committee and with the 1964 Helsinki declaration and its later amendments or comparable ethical standards.

Informed Consent: 

Written informed consent was obtained from the patient’s parents for publication of this case report and any accompanying images. 

## Case presentation

**Figure 1:**
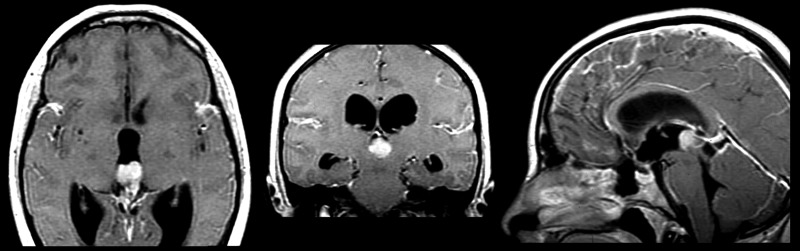
Pineoblastoma localized predominantly in posterior part of the third ventricle Secondary hydrocephalus is caused by obstruction of oral aperture of cerebral aqueduct

A 23-year-old female patient presented to Burdenko Neurosurgery Institute in the beginning of June 2010 complaining of headaches, nausea, vomiting, diplopia, and blurred vision. Informed patient consent was obtained for treatment. An MRI scan revealed a tumor of the pineal region and the posterior part of the third ventricle with secondary obstructive hydrocephalus (Figure 1).

**Figure 2:**
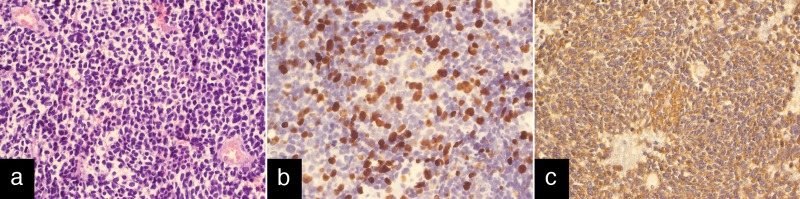
Histological images of primary tumor A: hematoxylin and eosin stain, x200. B: immunohistochemical study of Ki-67 (expression level was 20-25%), x200. C: expression of synaptophysin, x200

Neurological examination on admission revealed pretectal symptoms and bilateral papilledema. On June 16, the tumor was excised via a supracerebellar approach. The postoperative period was uneventful; the patient developed transient vertical gaze paresis. The histopathological study confirmed a pineoblastoma WHO Grade IV with its classic features: high cellularity with numerous mitotic figures, hyperchromatic nuclei with occasional small nucleoli, scant cytoplasm, and Homer-Wright rosettes. An immunohistochemical study demonstrated a strong expression of synaptophysin; the tumor was positive for NSE and NFP and negative for GFAP, PLAP, HCG, and AFP. Expression of proliferation marker MIB-1 (Ki-67) was 20-25% (Figure 2).

MRI scan of the spinal cord showed contrast enhancement of the spinal meninges, although no distinct metastatic lesions were found. In July and August of 2010, the patient underwent craniospinal irradiation (36 Gy in 20 fractions) with a 3D-conformal boost to the tumor bed (an additional 20 Gy in 10 fractions). From October 2010 until May 2011, chemotherapy was carried out with cisplatin, etoposide, and cyclophosphamide. After three cycles, cisplatin was substituted by carboplatin due to an elevated serum creatinine level. A total of five cycles of chemotherapy were accomplished. Follow-up MRI demonstrated the decrease of enhancement of the spinal meninges.

**Figure 3:**
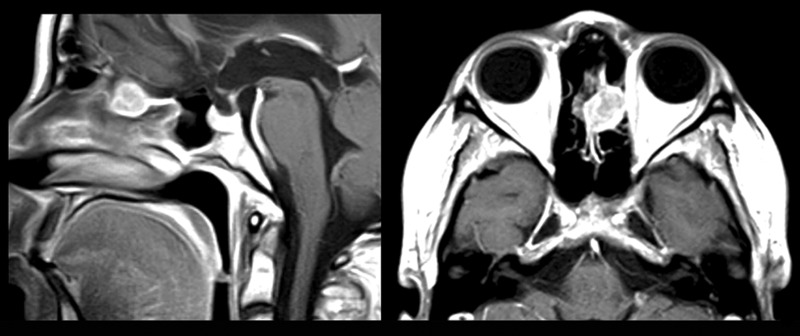
Preoperative MRI demonstrating lesion penetrating the anterior skull base Tumor involves left ethmoid roof and extends intracranially

7 months later (January 21, 2012), an MR study disclosed a lesion in the anterior skull base (Figure 3).

The patient was operated upon two months later using an endoscopic endonasal approach. The tumor was situated in the left ethmoid roof causing local destruction of the skull base with an invasion of the dura. En bloc resection was performed with exposure of the basal surface of the left frontal lobe. The skull base defect was reconstructed using two layers of fascia lata and fibrin glue. The sinonasal cavity was packed for four days and a prophylactic lumbar drain was placed for seven days. The patient was discharged without signs of CSF rhinorrhea and other complications. Typical postoperative nasal care was prescribed.

**Figure 4:**
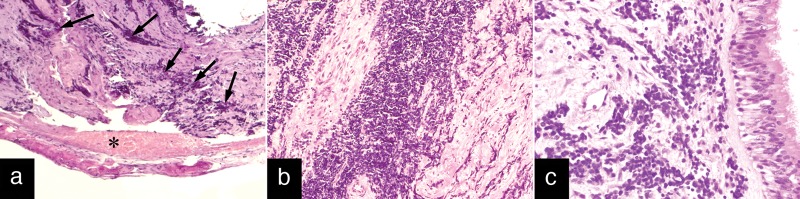
Histological images of metastatic tumor A: penetration of the ethmoid roof (asterisk) and invasion of the basal dura (arrows), hematoxylin and eosin stain, x100. B: masses of tumor invading the basal dura, hematoxylin and eosin stain, x200. C: invasion of sinonasal mucosa, hematoxylin and eosin stain, x400

Histological examination showed malignant primitive tumor with penetration of the bone and dural and mucosal invasion. Histopathological and immunohistochemical studies confirmed a primitive tumor of pineal parenchyma (pineoblastoma) and demonstrated a typical pattern of synaptophysin, NSE, and NFP (Figure 4). The cells of the tumor were negative for CD45, epithelial membrane antigen (EMA), GFAP, and pancytokeratin AE1/AE3.

One month later, hypofractionated stereotactic radiotherapy to the metastatic tumor bed was performed using the CyberKnife. A total dose of 30 Gy was delivered in five fractions. In December 2012, both the pineal region and anterior skull base were free from disease.

**Figure 5:**
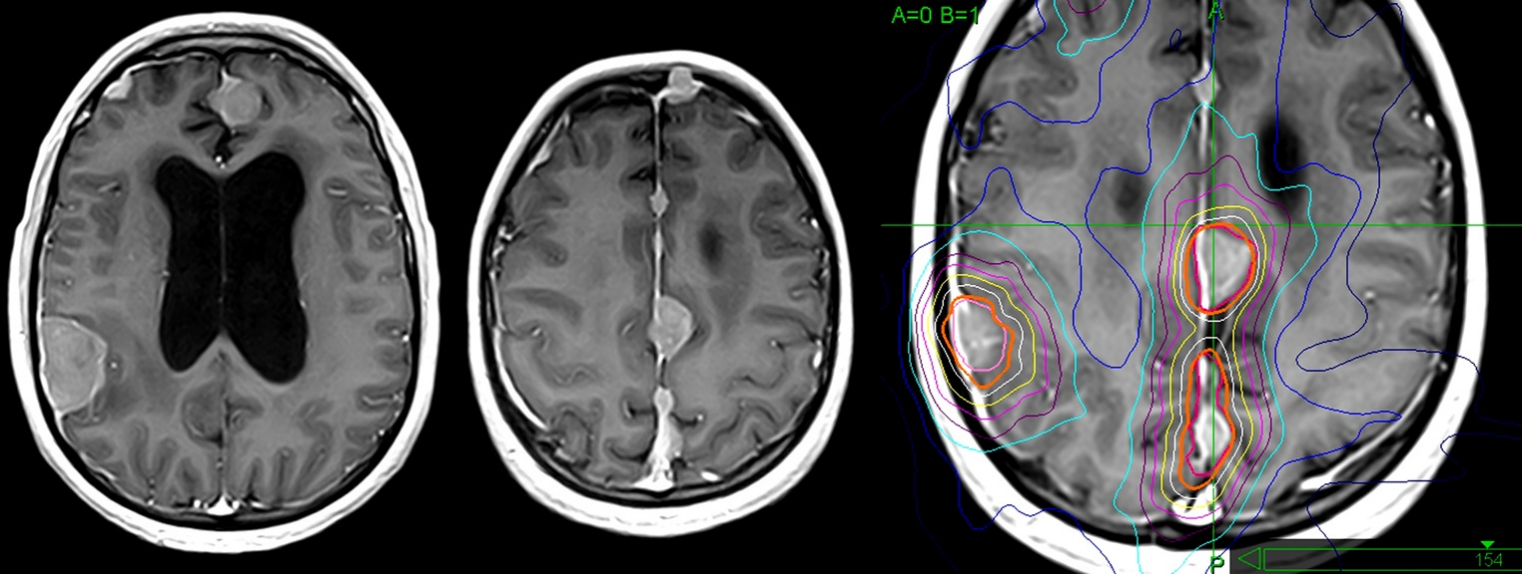
On MRI obtained 7 months after radiation treatment of skull base metastasis multiple leptomeningeal metastatic tumors are detected Left: pre-treatment MRI Right: dose plan of CyberKnife radiotherapy to the new leptomeningeal metastases (February, 2013) Lines: thick orange line - prescribed isodose, red and light pink lines - tumor contours. Isodose lines: white - 70%, yellow - 60%, magenta - 50%, purple - 40%, blue - 30%, dark blue - 10%

However, in February of 2013, over 12 intracranial leptomeningeal metastases were detected on MRI. The patient underwent second whole brain irradiation (20 Gy in 10 fractions) with radiosurgical boosts (single dose 13.4 – 13.6 Gy) to the three largest metastases (Figure 5).

An MRI in April of 2013 revealed complete regression of all foci. Despite this, four months later, one leptomeningeal metastasis of the frontal interhemispheric location recurred. This was treated with CyberKnife radiosurgery (single dose of 24 Gy). This frontal metastasis totally regressed, but at the same time, four foci (not irradiated with boost) relapsed. Etoposide chemotherapy was prescribed. At that time, MRI of the spine revealed no evidence of metastases.

**Figure 6:**
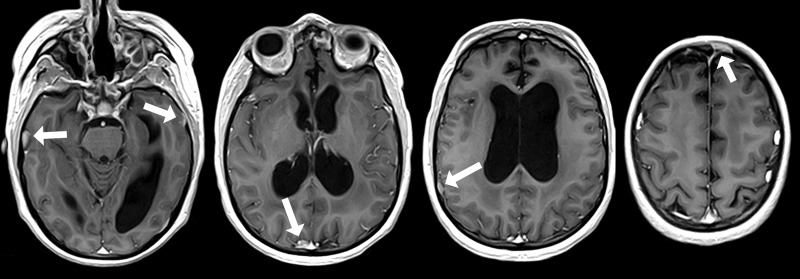
MRI in February, 2014 shows partial response of new leptomeningeal lesions to chemotherapy Residual foci are indicated by arrows

After three cycles of etoposide, the MRI showed a partial response of the intracranial lesions (Figure 6).

**Figure 7:**
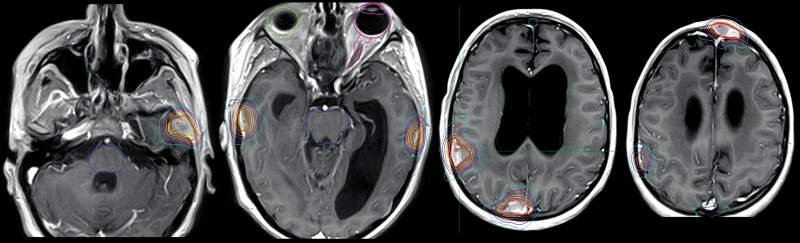
Dose plan of CyberKnife radiotherapy to new intracranial and intraosseous (frontal bone, left side) metastases (July, 2014) Lines: thick orange line - prescribed isodose, red and light yellow lines - tumor contours. Isodose lines: white - 70%, yellow - 60%, magenta - 50%, purple - 40%, blue - 30% Brainstem and eyeballs are delineated

By the next examination, after completion of six cycles of chemotherapy, some of the foci (not irradiated with boost) had progressed; therefore, etoposide chemotherapy was prolonged. Despite that, these active foci continued to grow. Etoposide was discontinued after eight cycles, and the patient underwent stereotactic radiosurgery to six intracranial lesions using the CyberKnife (single dose of 20 Gy to each) in July of 2014 (Figure 7). An extraneural metastatic lesion in the frontal bone was detected.

**Figure 8:**
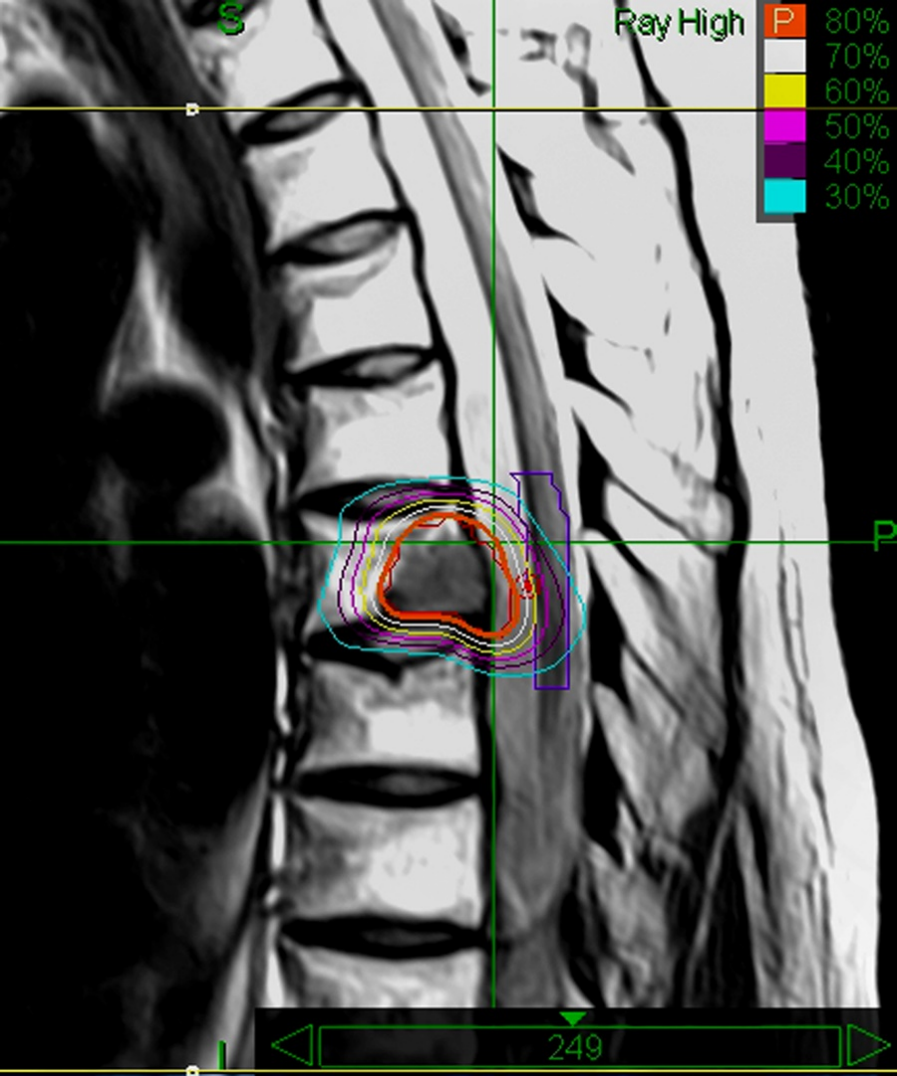
Dose plan of CyberKnife radiotherapy to the metastatic lesion of Th6 body Lines: thick orange line - prescribed isodose, dark red line - tumor contours. Isodose lines: white - 70%, yellow - 60%, magenta - 50%, purple - 40%, blue - 30%

In August of 2014, an MRI of the spine incidentally revealed a metastatic lesion of Th6 body, which was irradiated (24 Gy in three fractions) using the CyberKnife (Figure 8).

**Figure 9:**
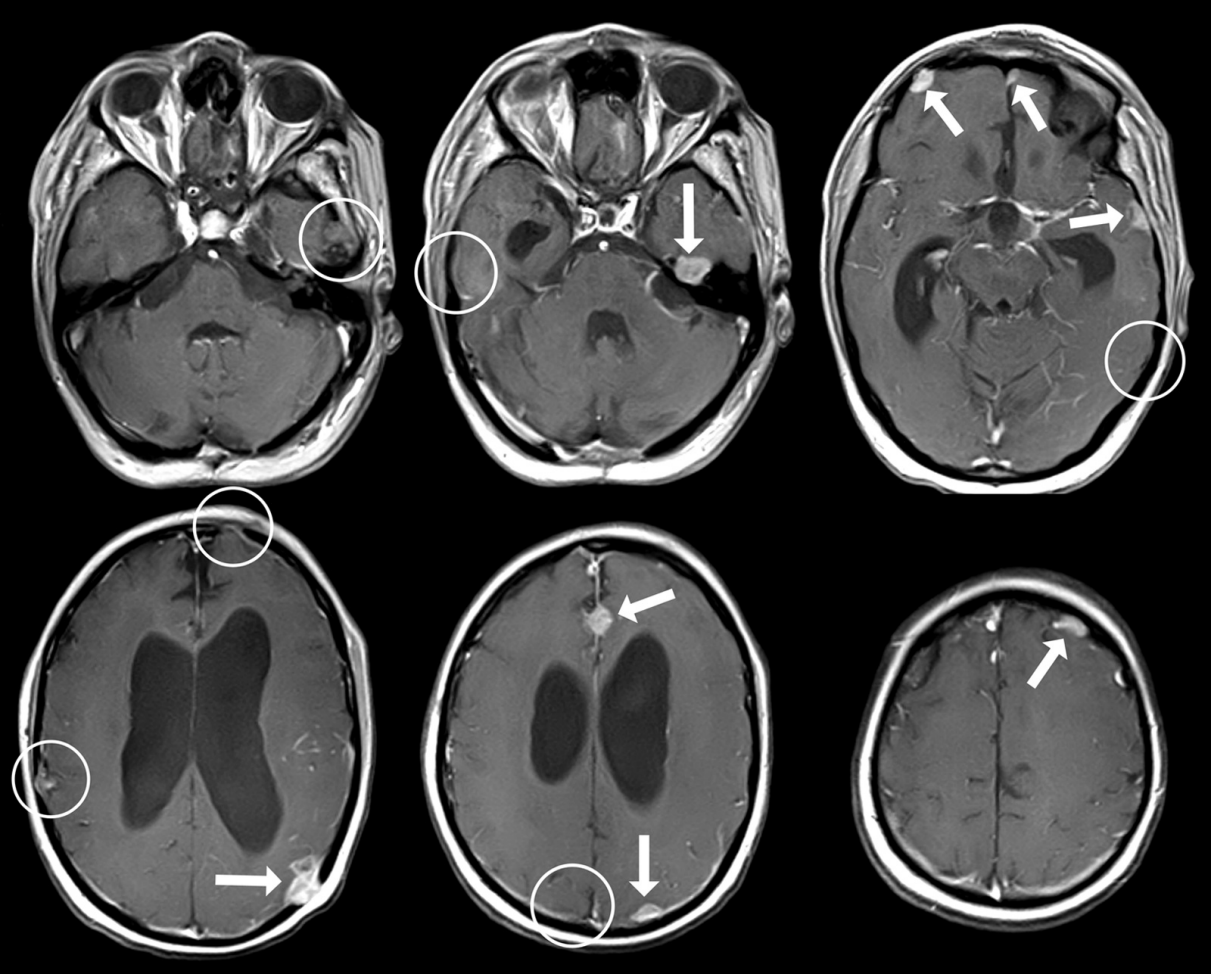
New leptomeningeal metastases (arrows), which appeared despite prolonged chemotherapy and two whole-brain radiation therapies Sites of previously irradiated metastases (July, 2014) are encircled

Three months later, the irradiated intracranial foci regressed except for one. In addition, new intracranial leptomeningeal metastases were found (Figure 9).

**Figure 10:**
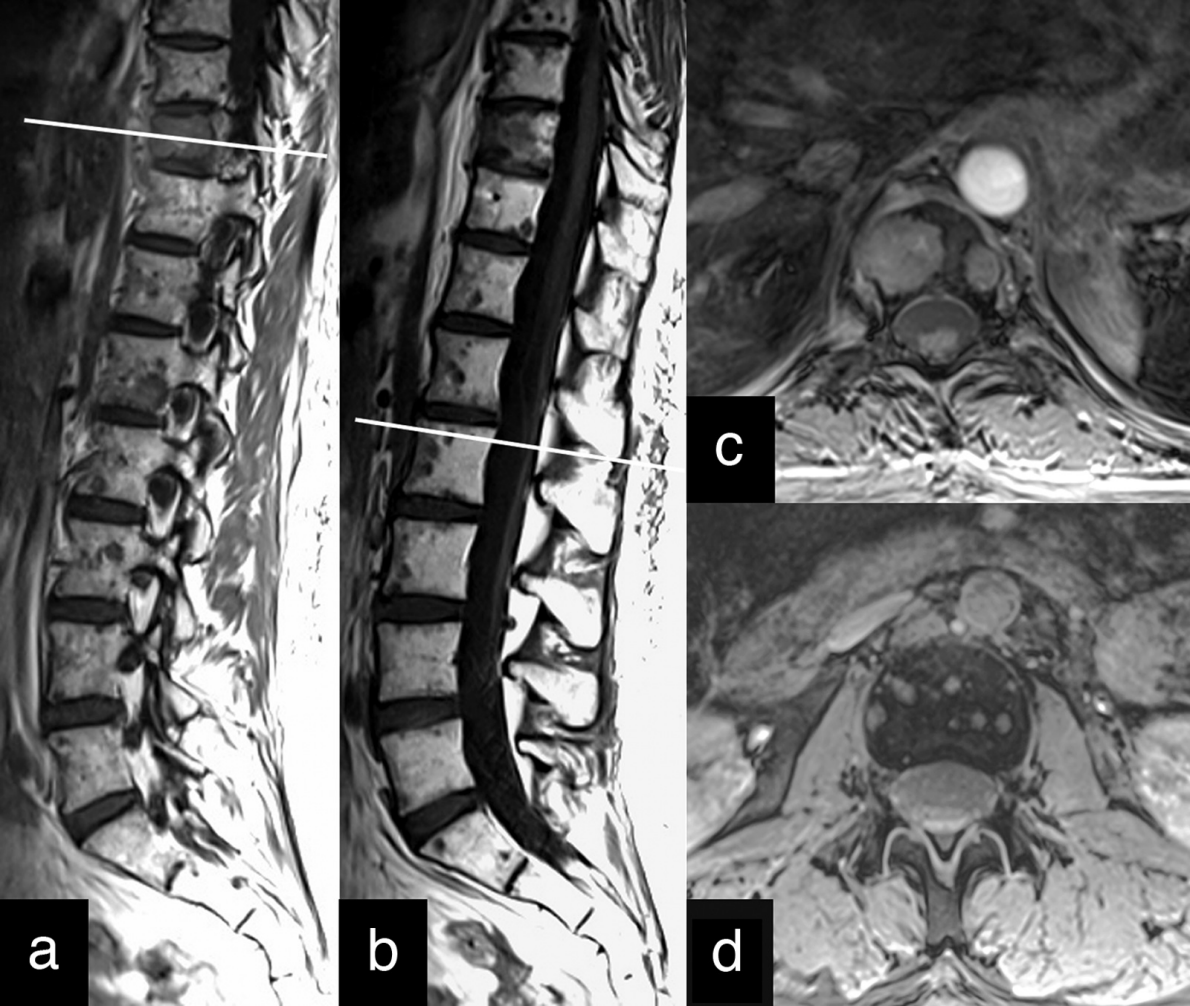
3-Tesla MRI of the spine discovers uncountable tiny metastatic lesions of the entire spinal column A: T1-weighted paramedian section. B: T1-weighted midsagittal section. C: T1-weighted axial section at the level indicated on a shows contrast enhancement of two gross metastases in Th10 vertebral body. D: T1-weighted axial section at the level indicated on B demonstrates multiple small metastases in L2 vertebral body

Shortly obtained last MRI showed not only new gross metastases in the Th10 and L1 vertebral bodies but also numerous small lesions in the entire spinal column (Figure 10).

The irradiated lesion of Th6 disappeared. There was no spinal cord or root deficit. Temozolomide was prescribed as salvage therapy. Despite treatment, the patient died at home .

## Discussion

Pineal tumors comprise 0.5-1% of all intracranial tumors, and 15-32% of pineal tumors arise from pineal parenchyma. Pineoblastoma represents 25-50% of pineal parenchymal tumors [1]. It is one of the most malignant lesions and is classified as a WHO Grade IV pineal parenchymal tumor. The prognosis in pineoblastoma is considered to be very poor: median survival time is 20 months, and the five-year survival rate is only 10% [4]. However, according to the recent single-center retrospective study of 31 patients with pineoblastoma published by Farnia, et al., median overall survival was 8.7 years with two-, five-, and 10-year actuarial rates of 89.5%, 69.4%, and 48.6%, respectively [5]. Being the PNET of pineal region, this tumor is characterized by aggressive clinical behavior and frequent leptomeningeal metastases along the craniospinal axis [2, 6].

Modern approaches to management of pineoblastoma include a combination of surgical resection (usually via a supracerebellar approach [1]), radiation therapy, and chemotherapy.

According to the earlier experience of Burdenko Neurosurgery Institute, dissemination of pineoblastoma after surgical removal of the primary tumor is associated with the absence of craniospinal irradiation and chemotherapy [7]. More recent modalities, such as radiosurgery, have been suggested as an adjunct to conventional radiotherapy or as a substitute for surgical resection, but convincing data is lacking [8].

Tate, et al. investigated outcomes by meta-analysis of 109 published studies (a total of 229 patients) [8]. Young age significantly predicted worse survival. Aggressive initial surgical resection improved survival. Univariate analysis demonstrated a significant association between treatment and survival: two-year survival rate was 35% after surgery and radiotherapy, 31% after surgery and chemotherapy, and 60% after surgery, radiation treatment, and chemotherapy. However, despite combined management, the frequency of metastases remains high. The authors showed that the rate of metastasizing after surgery, radiotherapy, and chemotherapy was 40%. The impact of disseminated disease at initial diagnosis was also studied. The two-year survival rate was 56% without dissemination and 37% in disseminated disease.

Interestingly, Farnia, et al. failed to find a correlation between age, gender, or extent of surgical resection with disease-free or overall survival; however, patients who received focal radiation initially experienced a higher rate of disease recurrence compared to those treated to the craniospinal axis [5].

Thus, the problem of metastases remains the cornerstone of treatment outcome. Besides well-known leptomeningeal dissemination, extraneural metastases are described. Constantine, et al. discriminated three possible routes of extraneural metastasizing: 1) surgical seeding of tumor cells beyond the dura and/or invasion of blood vessels with hematogenous spread, 2) through ventriculoperitoneal shunt, and 3) through Batson’s plexus (veins surrounding the vertebral column draining to the pelvic bones and upper femora) with subsequent hematogenous dissemination [3]. Data about metastasizing via ventriculoperitoneal shunt are controversial. Pitskhelauri, et al. described an observation of implantation metastases along the surgical approach after tumor removal [7].

Osseous metastases of pineoblastoma are the least common. Only six documented cases of osseous extraneural metastasis of pineoblastoma are reported in the literature (Table 1) [3].

**Table 1:** Reported cases of osseous extraneural metastases of pineoblastoma [3]

#	Authors and Year	Localization	Treatment and Outcome
1	Banerjee & Kak (1974)	Occipital bone, lungs and hilar lymph nodes	No treatment due to severe state of the patient
2	Jacobs & Rosenberg (1989)	Femoral bone	Palliative stabilization with a screw without known outcome
3	Fraser et al. (2000)	Acetabulum	Local irradiation (20 Gy), recurrence 7 months later, chemotherapy (6 months), involved field irradiation (36 Gy), subcutaneous nodules in the ipsilateral thigh 14 months later, 2 cycles of chemotherapy and electron beam irradiation (50 Gy), disease-free state for 3 years
4	Charafe-Jauffret et al. (2001)	Th8 vertebral body	Chemotherapy, relapse after 1 year, repeated cycles of chemotherapy, patient was alive and asymptomatic at the time of report
5	Charafe-Jauffret et al. (2001)	Sacrum (S4-S5)	Disease progression with meningeal, spinal and cranial invasion despite chemotherapy, death 2 years after sacral biopsy
6	Constantine et al. (2005)	Osseous and bone marrow metastases (pelvis, femur and ribs and vertebrae)	Regression of all metastatic lesions after chemotherapy carried out during a year

In our observation, intraosseous metastases (to the anterior skull base, calvarial bones, and then to the vertebral bodies) occurred after surgical resection, local radiotherapy combined with whole brain and spinal cord irradiation, and six cycles of chemotherapy.

We agree with Constantine, et al. [3] that when found in an isolated extraneural setting, osseous metastasis of pineoblastoma is a potentially curable situation. We demonstrated a good result of treatment of the single metastatic lesion in the ethmoid roof. Application of an endoscopic endonasal approach provided en-bloc resection with consistent reconstruction of the skull base defect. Our management tactics could be discussed in terms of the necessity of surgical resection. We performed an ethmoidectomy because histological verification was necessary to confirm the diagnosis. In our opinion, a locally aggressive osteolytic lesion carried a high risk of CSF rhinorrhea in the case of nonsurgical treatment. This was confirmed by alterations of the convexital intraosseous lesion of the frontal bone, which was irradiated, leaving a bone defect after complete regression (Figures 7, 9). Therefore, radical surgery with multilayer skull base reconstruction was undertaken.

The demonstrated case is the seventh published intraosseous metastasis of pineoblastoma and the first known description of metastasis in the anterior cranial base. Multiple intracranial metastases were suppressed using CyberKnife radiation treatment and chemotherapy until a massive involvement of the spinal column occurred. Our patient survived 57 months after primary disease manifestation and died of mushrooming metastases.

## Conclusions

Our observation demonstrates the possibility of local control of macroscopic disease using conventional and stereotactic radiotherapy, surgery, and chemotherapy. Two courses of whole brain radiation and prolonged chemotherapy led to only moderate neurocognitive decline. Interestingly, we did not observe any signs of brain radiation necrosis after repeated radiation treatments of numerous leptomeningeal metastases. Another issue that deserves special attention is the development of multiple intraosseous metastases (anterior skull base, calvarial bones and, finally, the entire spinal column) at the late stage of the disease. Unfortunately, the exact cause of lethal outcome is unknown, but we may suppose that the patient died from massive tumor dissemination.

## Appendices

### List of abbreviations

- AFP: alpha-fetoprotein
- CNS: central nervous system
- CSF: cerebrospinal fluid
- EMA: epithelial membrane antigen
- GFAP: glial fibrillary acidic protein
- HCG: human chorionic gonadotropin
- MRI: magnetic resonance imaging
- NFP: neurofilament protein
- NSE: neuron-specific enolase
- PLAP: placental alkaline phosphatase
- PNET: primitive neuroectodermal tumor
- WHO: World Health Organization
